# Telomere shortening and telomere position effect in mild ring 17 syndrome

**DOI:** 10.1186/1756-8935-7-1

**Published:** 2014-01-07

**Authors:** Cecilia Surace, Francesco Berardinelli, Andrea Masotti, Maria Cristina Roberti, Letizia Da Sacco, Gemma D’Elia, Pietro Sirleto, Maria Cristina Digilio, Raffaella Cusmai, Simona Grotta, Stefano Petrocchi, May El Hachem, Elisa Pisaneschi, Laura Ciocca, Serena Russo, Francesca Romana Lepri, Antonella Sgura, Adriano Angioni

**Affiliations:** 1 Cytogenetics and Molecular Genetics Unit, ‘Bambino Gesù’ Children’s Hospital, IRCCS, Piazza S. Onofrio 4, 00165 Rome, Italy; 2 Department of Biology, University ‘Roma Tre’, Rome, Italy; 3 Gene Expression-Microarrays Laboratory, ‘Bambino Gesù’ Children’s Hospital, IRCCS, Rome, Italy; 4 Medical Genetics Unit, ‘Bambino Gesù’ Children’s Hospital, IRCCS, Rome, Italy; 5 Neurology Unit, ‘Bambino Gesù’ Children’s Hospital, IRCCS, Rome, Italy; 6 Dermatology Unit, ‘Bambino Gesù’ Children’s Hospital, IRCCS, Rome, Italy

**Keywords:** Genetic syndrome, Telomere position effect, Ring 17 chromosome, Telomere shortening

## Abstract

**Background:**

Ring chromosome 17 syndrome is a rare disease that arises from the breakage and reunion of the short and long arms of chromosome 17. Usually this abnormality results in deletion of genetic material, which explains the clinical features of the syndrome. Moreover, similar phenotypic features have been observed in cases with complete or partial loss of the telomeric repeats and conservation of the euchromatic regions. We studied two different cases of ring 17 syndrome, firstly, to clarify, by analyzing gene expression analysis using real-time qPCR, the role of the telomere absence in relationship with the clinical symptoms, and secondly, to look for a new model of the mechanism of ring chromosome transmission in a rare case of familial mosaicism, through cytomolecular and quantitative fluorescence *in-situ* hybridization (Q-FISH) investigations.

**Results:**

The results for the first case showed that the expression levels of genes selected, which were located close to the p and q ends of chromosome 17, were significantly downregulated in comparison with controls. Moreover, for the second case, we demonstrated that the telomeres were conserved, but were significantly shorter than those of age-matched controls; data from segregation analysis showed that the ring chromosome was transmitted only to the affected subjects of the family.

**Conclusions:**

Subtelomeric gene regulation is responsible for the phenotypic aspects of ring 17 syndrome; telomere shortening influences the phenotypic spectrum of this disease and strongly contributes to the familial transmission of the mosaic ring. Together, these results provide new insights into the genotype-phenotype relationships in mild ring 17 syndrome.

## Background

Ring chromosomes are unusual circularized chromosomes, the vast majority of which arise sporadically [1]. Two types of ring chromosome can be related to different clinical presentations: the non-supernumerary ring, which replaces one of the normal homologs, with the karyotype 46,(r) and the supernumerary ring, which is often very small, with the karyotype 47,+(r). The non-supernumerary ring chromosomes are usually associated with abnormal phenotypes because of loss of genomic material at one or both ends. In some cases, no deletion has been detected and the phenotypic features have been attributed to mitotic ring instability [2].

The classic mode of ring chromosome formation consists of two terminal breaks in both chromosome arms, followed by a reconnection of the broken ends, leading to a loss of genetic material. Alternatively, they can be formed by fusion of subtelomeric sequences or telomere-telomere fusion with no deletion, resulting in complete ring chromosomes.

Telomeres, the nucleoprotein complexes that cap eukaryotic chromosomes, play two major roles in cell physiology. First, they are critical factors in determining the lifespan of mammalian cells. Second, they are essential for genome integrity maintenance. In addition to their role in protecting the ends of chromosomes, telomeres can influence the expression of nearby genes, an event called the telomere position effect. Previous reports demonstrated that the telomere position effect is an epigenetic process that involves changes in chromatin conformation and depends on both the distance from the telomere and the telomere’s length [3]. The telomere position effect also occurs in mammalian cells, and may play a role in human genetic disease, as a result of the repositioning of active genes near telomeres or subtelomeric sequences following chromosome rearrangements [4-8], such as those seen in individuals with ring chromosomes [9,10].

Rings of chromosome 17 are relatively rare and only 17 cases have been reported in the literature to date. Among these 17 cases, 11 showed no deletions of the Miller-Dieker critical region with mild phenotypic features [11] consisting of growth delay, intellectual disability, seizures, *café au lait* skin spots and minor facial dysmorphism.

The ring chromosomes 17 of the majority of these patients were described at the banding level, and only two cases pointed at the molecular level [11-13]. Cytomolecular investigation of the telomeric and subtelomeric regions of ring 17 is essential for the disclosure of candidate genes or gene expression regulating regions, which are potentially responsible for the phenotypic characteristics.

We report here the results of new investigations of a previously described patient with mild ring 17 syndrome and studies of an unusual case of ring chromosome 17 syndrome with a mosaic familial transmission.

## Results

### Case 1

#### Clinical presentation

The clinical features of patient 1 have been previously reported [11]. We evaluated the patient at four years of age. Clinical examination showed prominent eyes, epicanthal folds, anteverted nares, thick lips, small teeth, prognathism, generalized *café au lait* skin spots, and hypochromic cutaneous lesions. Developmental milestones were moderately delayed and language was absent. The patient developed epilepsy when he was two years old: this was characterized by tonic seizures that were difficult to control by medical treatment. The patient’s ductus arteriosus was closed by cardiac catheterization at five years of age. Cytogenetic investigations from peripheral blood cells revealed the following karyotype: 46,XY,r(17)(p13q25)[27]/45,XY,−17[3]. Chromosome analysis of fibroblasts obtained by dyschromic skin spot biopsy showed the sole ring 17 pattern without the monosomic cell line. Fluorescence *in-situ* hybridization (FISH) investigations revealed the loss of r(17) telomeres and conservation of the whole adjacent, euchromatic, subtelomeric regions.

#### Gene expression analysis

The expression levels of 24 selected genes within a 1.5 Mb region starting from each telomere and one housekeeping gene (*GAPDH*) of the ring 17 patient were measured in each sample by real-time qPCR and compared with controls. Two other genes (*NF1* and *LIS1*) that are located outside the 1.5 Mb region (17q11.2 and 17p13.3, respectively), were also considered. Of a total of 26 genes analyzed, 14 genes were statistically significant (*P* < 0.05), and 12 of these were located within the 1.5 Mb region (Table 1). Of these latter genes, seven were found to be deregulated by a fold change (FC) greater than 1.5: *RPH3AL*, *FAM57A*, *GEMIN4*, *NXN* in the sub-telomere region of 17p and *ARHGDIA*, *NARF*, and *FN3K* located in the 17q arm. The *FOXK2* gene displayed a downregulation that was below the FC threshold of 1.5 (FC = −1.44). The modulation of *YWHAE*, *MYOC1*, *SIRT7*, and *RNMTL1* is negligible, suggesting that these genes are not sensitive to the telomere influence. Interestingly, the *NF1* gene displayed a significant upregulation of 2.56, whereas *LIS1* is downregulated −1.76 fold. Taken together, these results emphasize the downregulation of the majority of genes included within the investigated sub-telomere regions.

**Table 1:** Expression levels of selected sub-telomere genes of the ring17 patient, significantly deregulated compared with controls

**Gene symbol**	**Gene name**	**Fold change**	***P***
		**(standard deviation)**	
**17p: 1 to 1,500,000**			
*RPH3AL*	Rabphilin 3A-like (without C2 domains)	−2.99 (0.15)	0.001
*FAM57A*	Family with sequence similarity 57, member A	−1.61 (0.04)	0.002
*GEMIN4*	Gem (nuclear organelle) associated protein 4	−1.70 (0.03)	0.001
*RNMTL1*	RNA methyltransferase like 1	1.13 (0.03)	0.018
*NXN*	Nucleoredoxin	−1.87 (0.05)	0.001
*YWHAE*	Tyrosine 3-monooxygenase/tryptophan 5-monooxygenase activation protein, epsilon polypeptide	−1.04 (0.01)	<0.001
*MYO1C*	Myosin IC	−1.15 (0.01)	0.001
**17q: 79,695,210 to- 81,195,210**			
*ARHGDIA*	ρ GDP dissociation inhibitor α	−1.67 (0.02)	<0.001
*SIRT7*	Sirtuin (silent mating type information regulation 2 homolog) 7 (*S. cerevisiae*)	−1.09 (0.01)	0.010
*NARF*	Nuclear prelamin A recognition factor	−1.86 (0.02)	<0.001
*FOXK2*	Forkhead box K2	−1.44 (0.07)	0.008
*FN3K*	Fructosamine 3 kinase	−2.18 (0.06)	<0.001

Genes are listed from the telomere down to 1.5 Mb on the p arm and from the position 79,695,210 of the q arm up to the telomere. The expression values are reported as the mean fold change of three replicates.

### Case 2

#### Clinical presentation

The patient, a girl, was the first child of unrelated parents. At birth, the mother was 27 years old and the father was 29. The baby was born at term by vaginal delivery after an uneventful pregnancy. The baby’s birth weight was 3100 g, she was 48.5 cm long, and the circumference of her head was 33.5 cm. The patient was first evaluated at 2.4 years of age. Her weight was 12 kg (25th centile), she was 79 cm (<3rd centile) high, and her head circumference was 45 cm (<3rd centile). Clinical examination showed sparse *café au lait* spots on her trunk and legs, as in her mother. No facial anomalies were noted. Developmental milestones were mildly delayed (sitting at nine months, walking alone at 18 months). At six years of age, an electroencephalogram (EEG) was conducted because of nocturnal episodes of generalized stiffness, demonstrating interictal epileptic-form abnormalities. The ictal EEG revealed generalized tonic seizures. Adrenocorticotropic hormone was able to reverse the epileptic encephalopathy. However, two months later, the seizures appeared again and the child is actually severely refractory now. Seizures were difficult to control with topiramate, lamotrigine, or levetiracetam. Magnetic resonance imaging findings were normal. An ophthalmological examination showed a flecked retina; electroretinogram and visual evoked potentials were normal. The first cognitive evaluation detected a borderline cognitive level. However, when the child was eight years old, her EEG and clinical pattern suggested epileptic encephalopathy, and the child experienced cognitive deterioration.

The mother’s child had multiple *café-au-lait* spots on her trunk without facial anomalies or cognitive deficit.

#### Classical and molecular cytogenetic investigations

**Figure 1:**
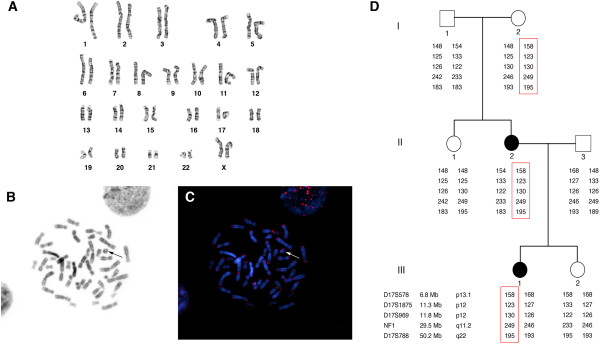
**Cytomolecular results obtained from patient 2. (A)** G-banded karyotype. **(B,C)** Representative images of a metaphase stained for telomere sequences and chromosome 2 centromere in **(B)** grayscale and **(C)** colour. Arrow indicates ring chromosome. **(D)** Pedigree of patient’s family. Informative short tandem repeats (STRs) are listed on the left and the size and chromosome position of each STR is reported. The haplotype passing from the maternal grandmother (I-2), to the mother (II-2) and to the proband (III-1) is highlighted with a red rectangle.

A study of the lymphocytes revealed a mosaic karyotype: 46,XX,r(17)(p13q25)[36]/46,XX[14] (Figure 1A). G-banding analysis of the fibroblasts showed two cell lines, the most abundant clone having a normal karyotype (98%) and the other having a double ring 17 (2%). Chromosome analysis of the patient’s parents displayed a normal paternal karyotype, and a mosaic ring 17 maternal karyotype: 46,XX,r(17)(p13q25)[22]/46,XX[28]. All the FISH pan-telomeric (Figures 1B,C) and subtelomeric probes showed clear signals in the patient and her mother, both on the normal chromosome 17 and on the ring. This confirmed that no terminal deletion had occurred. An array-CGH study of both cases did not detect any submicroscopic deletions or duplications. Segregation analysis of the family group revealed that the ring of the patient and her mother derived from the chromosome 17 of the patient’s maternal grandmother (Figure 1D). The same study also excluded uniparental disomy because all of the informative markers were found on both the normal and the rearranged chromosomes. Additional microsatellite investigation, performed on fibroblasts of normal skin and a *café* au lait skin spot biopsy of the patient, displayed the presence of a biallelic pattern of transmission related to each parent (Table 2).

**Table 2:** Segregation analysis of the fibroblasts of the patient

	**D17S1839**	**D17S1301**	**D17S752**	**D17S788**
III-1 normal and *café au lait* skin spot biopsy	234/242	148/153	162/171	195/193
II-2 maternal peripheral blood	242/245	148	162	183/195
II-3 paternal peripheral blood	234/252	153/157	158/171	189/193

These data clearly show that the proband III-1 inherited one chromosome 17 from her mother (II-2) and the other one from her father (II-3).

#### Quantitative fluorescence in-situ hybridization (Q-FISH) analysis

**Figure 2:**
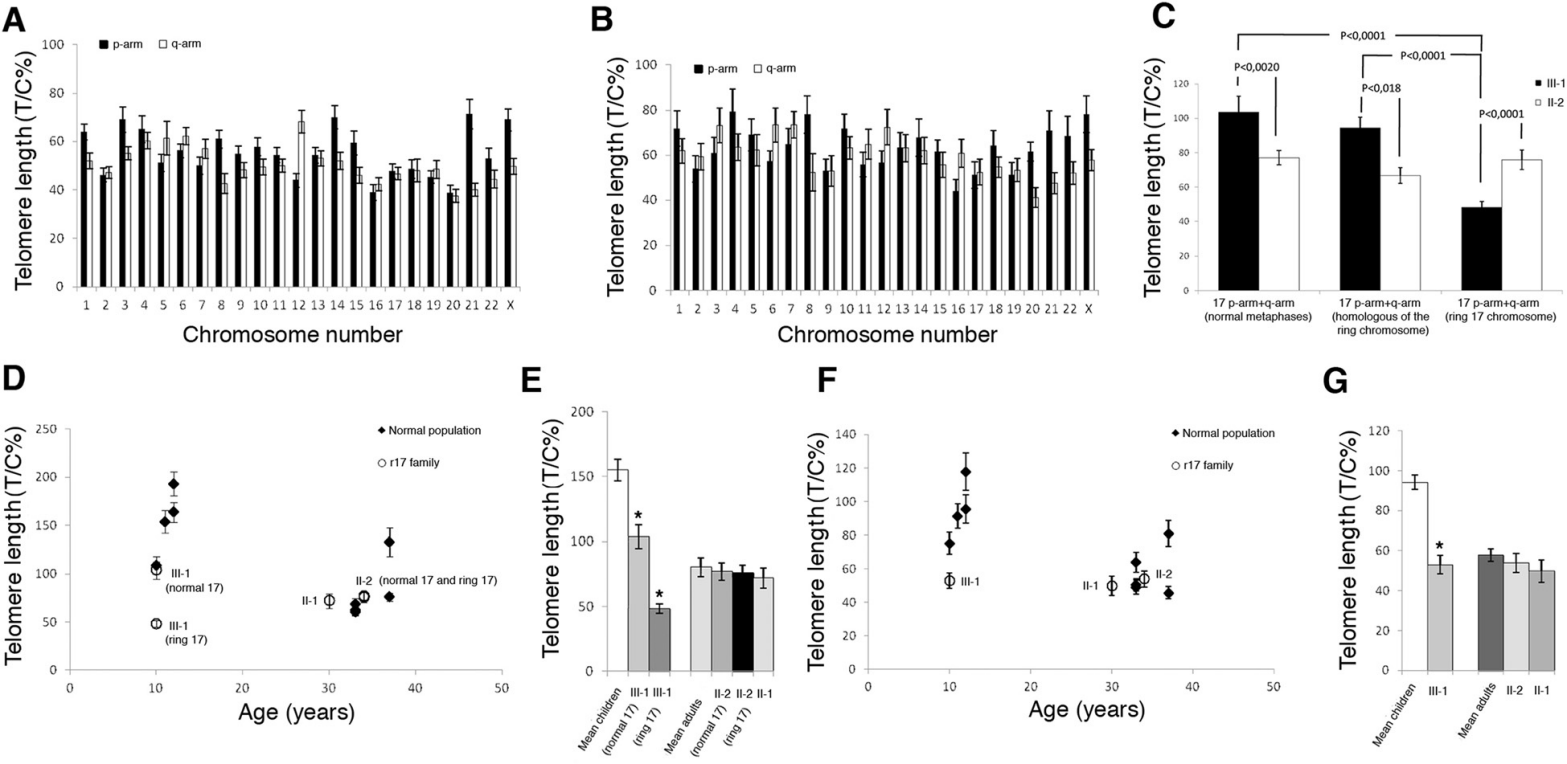
**Quantitative fluorescence *in-situ *hybridization (Q-FISH) results. (A,B)** Chromosome-specific telomere lengths, as evaluated in **(A)** normal and **(B)** ring metaphases in lymphocytes established from the proband (III-1). Chromosome 17 telomere lengths in ring metaphases refer solely to the homolog of the ring chromosome. **(C)** Average telomere length in ring chromosome, in the homolog of the ring chromosome and in normal chromosomes 17 from normal metaphases (p-ter + q-ter), as addressed by Q-FISH analysis in lymphocytes established from proband (III-1) and her mother (II-2). **(D,E)** Comparison between chromosome 17 telomere length (p-ter + q-ter) in r17 family members and age-matched controls. Control values were represented as **(D)** independent or **(E)** averaged values. **(F,G)** Comparison between the mean cellular telomere length in r17 family members and age-matched controls. Control values were represented as **(F)** independent or **(G)** averaged values.

Chromosome-specific telomere length was assessed in either normal (Figure 2A) or ring metaphases (Figure 2B) obtained from the patient. Data showed that the means of all telomere lengths in normal and ring metaphases (61 and 53 T/C% respectively) did not differ significantly. Moreover, the telomere length patterns in the two metaphase classes were very similar, and in both the shortest telomeres were located at 20q, 16p, 21q, 22q, 19p, 17p, and 17q whereas the longest were located at Xp, 4p, 21p, 12q, 8p, 14p, 1p, and 6q.

Telomere length analysis of chromosome 17 in the patient showed that telomere length in the ring chromosome (48 T/C%) was significantly shorter than the sum of the telomeres at the p-ter and the q-ter in normal chromosome 17, if scored in normal (104 T/C%) or ring metaphases (95 T/C%) (Figure 2C). Conversely, the telomeric DNA in the ring chromosome of the patient’s mother (76 T/C%) was comparable in length to that of the normal chromosome 17 in normal metaphases (77 T/C%) and in ring metaphases (67 T/C%) (Figure 2C). As expected, telomeres in the normal chromosome 17 were longer in the patient than in her mother, while telomeres in the ring chromosome were significantly shorter in the patient than in her mother (48 T/C% and 76 T/C% respectively) (Figure 2C).

To compare the ring 17 of the family to those of normal healthy individuals, lymphocytes from nine (four children and five adults) normal donors were also analyzed for telomere length (Table 3). Considering the mean cellular telomere length, we found an age-related telomere erosion in the control individuals, with the mean telomere lengths ranging from 94 T/C% in children to 58 T/C% in adults (Figure 2F,G). The values for the patient’s mother and aunt (54 and 50 T/C% respectively) were comparable to the age-matched controls (58 T/C%), indicating a normal telomere length in both family members. Interestingly, the patient showed reduced telomeric lengths with telomeres much shorter (53 T/C%) than the other age-matched children (94 T/C%) (Figure 2F,G).

**Table 3:** Telomere lengths in members of the ring 17 family and in normal age-matched individuals

	**Age (years)**	**Cell scored**	**Mean cellular telomere length**	**Normal metaphases**	**Ring metaphases**	
				**17p arm +17q arm**	**17p arm +17q arm (homolog of ring chromosome)**	**17p arm +17q arm (ring chromosome)**
**III-1**	10	51*	53.0 ± 4.6	103.7 ± 9.2	94.5 ± 6.3	48.1 ± 5.6
**cntr1**	10	20	75.1 ± 6.5	108.9 ± 8.9		
**cntr2**	11	20	91.4 ± 7.4	153.9 ± 11.8		
**cntr3**	12	20	117.8 ± 11.3	193.3 ± 16.5		
**cntr4**	12	20	95.5 ± 8.4	163.7 ± 14.1		
**II-1**	30	19	49.9 ± 5.6	71.9 ± 7.7		
**II-2**	34	68**	54.0 ± 4.8	77.0 ± 4.2	66.8 ± 4.6	75.9 ± 5.6
**cntr5**	33	20	50.5 ± 3.6	68.7 ± 6.0		
**cntr6**	33	20	63.8 ± 6.0	62.7 ± 6.1		
**cntr7**	33	20	49.1 ± 4.3	60.7 ± 3.6		
**cntr8**	37	20	45.7 ± 3.6	76.0 ± 4.2		
**cntr9**	37	20	80.9 ± 7.7	132.7 ± 14.9		

Data are shown for all the individuals analyzed. *18 normal metaphases and 33 carrying the ring 17 chromosome. **46 normal metaphases and 22 carrying the ring 17 chromosome.

When we turned our attention to chromosome 17 telomere lengths (ring and normal), we observed the same trend in mean cellular telomere length. Thus, the patient displays telomeres on chromosome 17 (both ring and normal) shorter than those of normal healthy children, whereas the other members of the family were not significantly different in telomere length, as compared with age-matched controls (Figure 2D,E).

## Discussion

Ring chromosomes are rare cytogenetic abnormalities that usually occur as a consequence of breaks and deletions at both ends of the chromosome, with subsequent fusion of the remaining portions. The breakpoints may be located within euchromatic regions, with partial monosomy of the distal ends of the short and long arms of the chromosome, but sometimes they may involve only one or both the telomere repeats. Though uncommon, the last mechanism has been described in the literature as a telomere-telomere fusion with no detectable loss of euchromatin [14,15].

In a previous report [11], we have described the case of a ring 17 chromosome in a young boy, providing an accurate characterization of the defect, which resulted in complete loss of the telomeric ends with the conservation of the whole euchromatic regions. The clinical picture was reported as a mild ring 17 syndrome with moderate intellectual disability, seizures, dysmorphic traits, and *café au lait* and hypochromic skin spots. We compared this to the most severe lissencephaly syndrome, which involves functional loss of the *LIS1* gene.

Several hypotheses have been proposed to explain the relevant phenotype of the mild ring chromosome 17 syndrome, such as ‘dynamic mosaicism’ or the ‘ring syndrome’. However, they have never been supported by clear experimental evidence. It has been suggested that the epigenetic silencing of gene expression through a telomere position effect is one of the alternative mechanisms that occur in ring chromosome syndrome [9]. This silencing effect contributes to an alteration of the function of genes located near the ring fusion point by modulating (mainly downregulating) their expression in a telomere-dependent fashion [10,16-18].

Here, we note that, in the absence of detectable telomere repeats in the first patient [11], a group of non-contiguous genes that were within an interval of 1.5 Mb from the telomeres of p and q arms were significantly downregulated compared with the controls. Although the biological functions of these genes are manifold, most of the latter play a key role in cellular and tissue functions (that is, *RPH3AL, FN3K*, and *NARF*), cell cycle (that is, *ARHGDIA*), DNA processing and regulation (that is, *FOXK2* and *GEMIN4*) and cell growth and early development (that is*, NXN* and *FAM57A*). Our data suggest that the concomitant downregulation of these genes may lead to an imbalance of cell activities and hamper proper physiological interactions, ultimately producing the phenotypic aspects related to ring 17 syndrome. Moreover, the upmodulation of *NF1* and the downregulation of *LIS1*, located outside the studied 1.5 Mb region, reveal that the observed effects are not linearly correlated to the distance from the telomeres.

The second case we studied regards a young girl with a cytogenetic finding of mosaic ring 17 chromosome. The clinical phenotype presents, as in the previous case, the classical features of mild ring 17 syndrome, with the key symptoms being seizures, intellectual disability, and *café au lait* skin spots. Nevertheless, chromosome and molecular cytogenetic investigations demonstrated that the telomere ends were preserved. Familial studies revealed that the mother was the carrier of a mosaic ring 17 chromosome with the sole phenotypic feature of *café au lait* skin spots. Ring chromosomes usually follow the conventional method of transmission resulting, when the rearrangement is passed on, in a constitutional ring syndrome with all, or the majority, of cells containing the abnormal chromosome. The familial transmission of a mosaic ring is extremely rare and represents a challenge still to be clarified [1]. The proband’s maternal grandparents, sister, and aunt exhibited a normal karyotype in accordance with the data of the segregation analysis that clearly showed that the ring chromosome derived from the maternal grandmother and was transmitted only to the affected subjects. We also excluded uniparental disomy, since all of the informative markers were found on both the normal and the rearranged chromosomes. Moreover, we tested a set of microsatellites on normal skin and a *café au lait* skin spot biopsy of the patient, and this showed the presence of a biallelic pattern of transmission related to each parent.

Analysis of telomere lengths in the patient’s lymphocytes revealed that telomeres of chromosome 17 were the shortest compared with the other chromosomes of the cell in both normal and ring metaphases (Figure 2A,B). This evidence raises the hypothesis that chromosome 17 could be prone to reaching critical telomere lengths, leading to an increased risk of ring formation.

When we compared the telomere lengths of the ring chromosome of the patient and her mother with the mean telomere lengths of chromosome 17 in age-matched controls, we found that telomeres of the ring chromosome of the patient were at least three times shorter (48 T/C% vs. 155 T/C%) (Figure 2D,E), whereas no remarkable differences were found for the patient’s mother (76 T/C% vs. 80 T/C%). Therefore, assuming that the ring chromosome is generated during prenatal life and that ring chromosome telomeres do not shorten upon cell divisions (as telomeres usually do), we can hypothesize that the generation of the ring chromosome is the result of an extensive loss of telomere repeats at an early stage of development.

The mosaicism rate is another important aspect to consider in determining the phenotypic features of the syndrome. Values less than 10% do not usually present clinical manifestation, whereas higher rates are associated strictly with different degrees of disease expression. Conversely, this rate tends to decline through one’s lifespan. In fact, the rearranged chromosome is preferentially lost during cell divisions, leading to apoptosis and premature cell death. In our case, the patient and her mother showed respectively 72% and 44% of mosaic cells in the peripheral blood. Nevertheless, only the patient presented relevant symptoms. The telomere shortening observed in the patient with respect to the mother appears as an additional factor that might have influenced the generation of the ring chromosome and the occurrence of this peculiar phenotype. These hypotheses are also confirmed by the first case that we investigated, which completely lacked the telomeres, as well as previous reports describing ring chromosome 17 with subtelomeric deletions, but conserving the *LIS1* gene.

## Conclusions

In summary, our studies clearly outline that telomere length reduction plays a determining role in at least two important aspects of the ring 17 syndrome.

First, the cases we presented show a strong genotype-phenotype correlation regarding clinical symptoms and telomeric shortening. Patient 1, with the most severe phenotype, lost the telomeric ends completely. Patient 2, with an intermediate picture, displayed a high shortening of the telomeric repeats, while her mother, affected by a lower reduction of telomere length, presented with only skin spots without any other symptom.

Second, telomere shortening acts as a novel recognized mechanism responsible for ring formation and mosaicism assessment. In fact, we demonstrated that the ring chromosome was not selectively lost during the cell cycle, being the autologous maternal linear chromosome present on all cells in the skin biopsy. Moreover, the ring chromosome of the patient’s mother, being unable to change its structural characteristic during gametogenesis, has telomeres longer than that of the patient. These observations led us to hypothesize that the chromosome transmitted is the normal autologous one and that, as a consequence of the telomere shortening during embryogenesis, it underwent a circularization process, ultimately leading to the formation of a ring chromosome. The stage in which this occurrence takes place is responsible for the different mosaicism rate.

The influence of telomere shortening and the telomere position effect should also be investigated in other patients carrying the ring chromosome 17; however, since the ring chromosome 17 syndrome is very rare, a big effort by all the scientific community will be necessary to recruit a larger number of patients.

## Methods

### Gene expression analysis

All the samples were collected according to the current version of the Declaration of Helsinki and written informed consent for publication of this manuscript and accompanying images was obtained from the patients’ parents and relatives. All experiments were ethically approved by the ‘Bambino Gesù’ Children’s Hospital Scientific Board.

Skin fibroblasts of the patient were obtained from a biopsy of a hypochromic cutaneous lesion using standard methods. Skin fibroblasts of two healthy children were also collected and used as calibrator samples (reference). Total RNA was isolated from fibroblasts with TRIzol (Ambion, Life Technologies, Paisley, UK) according to the manufacturer’s protocol. For qPCR analysis, 1 μg of total RNA was reverse-transcribed to cDNA using a high-capacity cDNA archive kit (Applied Biosystems, Life Technologies, Paisley, UK). The expression levels of 26 selected genes and one housekeeping gene (*GAPDH*) of the ring17 patient were measured in each sample by real-time qPCR using pre-designed TaqMan gene expression assays on an ABI PRISM 7900HT Sequence Detection System (Applied Biosystems) according to the manufacturer’s protocol, and compared with controls.

The investigations involved 15 genes (*ACTG1, GCGR, ARHGDIA, NPB, SIRT7, SECTM1, UTS2R, NARF, FOXK2, RAB40B, FN3KRP, FN3K, TBCD, ZNF750, METRNL*) and 9 genes (*DOC2B, RPH3AL, FAM57A, GEMIN4, RNMTL1, NXN, TUSC5, YWHAE, MYO1C*) located on the sub-telomere region of the long and short arms, respectively, of chromosome 17 encompassing a 1.5 Mb region, starting from each telomere. Two other genes (*NF1* and *LIS1*) placed outside the 1.5 Mb region (17q11.2 and 17p13.3, respectively), were also investigated.

For each sample, three replicates were run for each gene in a 96-well plate. Real-time qPCR reactions were carried out following the manufacturer’s instructions. Gene expression values were determined as ΔCt (Ct(GAPDH) − Ct(gene)) and relative quantities between different samples were determined as ΔΔCt (ΔCt(patient) − ΔCt(calibrator sample)) [19]. All data are expressed as mean ± standard deviation. The statistical comparison between the ring 17 patient and control individuals was performed using Student’s *t* test at a level of significance of 0.05 (*P* < 0.05).

### Classical and molecular cytogenetic investigations

Karyotyping with G-banding was performed using standard methods on lymphocytes obtained from peripheral blood and fibroblasts from a biopsy of skin with or without hypochromic cutaneous lesions. Chromosomes were also examined on lymphocytes of both of the patient’s parents, as well as her sister, her maternal grandparents and her aunt. The karyotypes were described according to the International System for Human Cytogenetic Nomenclature (ISCN 2009).

To search for possible deletions at telomeric regions, FISH was performed with human pan-telomeric probes, P1 artificial chromosome (CTD-2348K1, 17p13.3) and fosmid (WI-837H17, 17q25.3) clones. The probes were directly labeled with Cy3-dUTP and fluorescein-dUTP (Perkin Elmer Life Sciences, Boston, MA, USA).

Array-CGH was performed using Agilent Technologies Array-CGH Kits (Santa Clara, CA, USA), as described previously [20]. The platform is a 60-mer oligonucleotide-based microarray with an overall median probe spatial resolution of 13 kb. To evaluate whether the copy number variations, detected by array-CGH, were potentially correlated with the clinical phenotype of our patient, bioinformatic analysis was carried out, consulting the Database of Genomic Variants BioXRT (http://projects.tcag.ca/variation/).

### Segregation analysis

DNA was extracted from peripheral blood of the patient and her relatives with a high pure PCR template preparation kit (Roche, Mannheim, Germany), according to the producer’s instructions. To assess the familiar origin of the ring 17, a panel of STRs was assembled using multiple primer pairs obtained from the UniSTS database (http://www.ncbi.nlm.nih.gov/unists/). The STRs analyzed, listed starting from 17pter to 17qter, were: D17S578, D17S1875, D17S969, NF1, D17S788, D17S1306, D17S1819, D17S789, D17S1839, D17S1292. The first five STRs were the informative ones. DNA was amplified by means of a GeneAmp PCR System 2700 (Applied Biosystems, Foster City, CA, USA) following the standard protocol. One primer from each pair was fluorescently labeled and PCR products were run on an ABI Prism 310 (Applied Biosystems), using GeneMapper v 3.0 software.

Additional studies were performed also on *café au lait* skin spots and a normal skin biopsy of the patient to investigate the parental transmission of the chromosome 17.

### Q-FISH analysis

Q-FISH staining was performed as previously described [21] with minor modifications. Briefly, 48 hours after the seeding, slides were rinsed with PBS at pH 7.5, and fixed in 4% formaldehyde for 2 min. After two rinses in PBS, the slides were incubated in acidified pepsin solution for 10 min, rinsed, and dehydrated through graded alcohols. Slides and probes (Cy3 linked telomeric and chromosome 2 centromeric PNA probe, DAKO Cytomatation, Denmark) were co-denatured at 80°C for 3 min and hybridized for 2 hours at room temperature in a humidified chamber. After hybridization, slides were washed twice for 15 min in 70% formamide, 10 mM Tris at pH 7.2, and 0.1% BSA, followed by three 5-minute washes in 0.1 M Tris at pH 7.5, 0.15 M NaCl, and 0.08% Tween 20. Slides were then dehydrated with an ethanol series and air dried. Finally, slides were counterstained with 4,6-diamidino-2-phenylindole (Sigma Aldrich, St. Louis, MO) in Vectashield (Vector Laboratories, Burlingame, CA). Images were captured at 63× magnification with an Axio Imager M1 (Carl Zeiss, Germany) equipped with a charge-coupled device camera, and the telomere size was analyzed with ISIS software (MetaSystems, Germany). The software calculates telomere lengths as the ratio between the fluorescence of each telomere signal and the fluorescence of the centromere of chromosome 2, used as the internal reference in each metaphase analyzed. The centromere 2 DNA sequence, which the probe recognizes, has a stable length and can be used as a reference. Data were expressed as a percentage (T/C%) [22]. For each individual, at least 20 metaphases have been analyzed.

## Abbreviations

BSA: Bovine serum albumin; EEG: Electroencephalogram; FC: Fold change; FISH: Fluorescence *in-situ* hybridization; PBS: Phosphate-buffered saline; PCR: Polymerase chain reaction; Q-FISH: Quantitative fluorescence *in-situ* hybridization; qPCR: Quantitative polymerase chain reaction; STR: Short tandem repeat.

## Competing interests

The authors declare that they have no competing interests.

## Authors’ contributions

CS, MCR, PS, LC, and SR performed biological sample processing and cytomolecular investigations. FB and AS carried out Q-FISH analysis. AM and LDS performed gene expression studies. GD, SG, SP, and EP carried out segregation analysis and interpreted the data. MCD, RC, and MEH recruited patients, collected biological samples, and performed clinical evaluations. CS, FB, AM, MCR, LDS, AS, and AA analyzed the data and drafted the manuscript. FRL critically reviewed the manuscript. CS, MCR, and AA conceived and designed the study. All authors read and approved the final manuscript.
